# Physical Exercise Interventions for Drug Addictive Disorders

**Published:** 2017-05-29

**Authors:** Trevor Archer, Rajendra D. Badgaiyan, Kenneth Blum

**Affiliations:** 1 Department of Psychology, University of Gothenburg, Gothenburg, Sweden; 2 Department of Psychiatry, Wright State University School of Medicine, Dayton, OH, USA; 3 Departments of Psychiatry and Behavioural Sciences, Keck School of Medicine of USC, Los Angeles, CA, USA

Physical exercise physical and psychological health positive through various different avenues, as example, through affecting positively cognitive performance based upon the relocation of cortical activity which seems to advancing the brain development, connectivity and resilience [1]. Any bodily activity that enhances or maintains physical fitness implies the engagement of regular and frequent exercise thereby maintaining physical fitness and the reduction of agents associated with health problems, e.g. cortisol. With regard to the large proportion of individuals with more-or-less sedentary occupations, physical exercise offers probably the most effective health-promoting lifestyle available with positive outcomes for both neurologic and psychiatric conditions [2–10]. The expressions of disorder emerging as consequences of exposure to reward loss have been neglected in approaches to the psychobiology of substance abuse disorders. This notion emphasizes the shared characteristics reward loss and addiction are reviewed, namely, the neural circuitry involved in reward devaluation, the influence of genetic and reward history on the behavioral vulnerability and resilience, the role of competing natural rewards, and emotional self-medication as a backdrop [11] to the consequences evolving in the “Reward Deficiency Syndrome”. The Reward Deficiency Syndrome, characterized by expressions of reward-seeking behavior and/or addictions and involving a G protein-coupled receptor located on postsynaptic dopaminergic neurons that is centrally involved in reward-mediating mesocorticolimbic pathways, originates from genetic variations, most notably resulting from those carrying the D2A1 allele implicated in addiction and abuse [12, 13]. Individuals carrying the A1 allele tend to have insufficient numbers of D2 receptors in their brain, resulting in lack of pleasure and reward from activities that would provide others with pleasure. Dopamine subtype 2 receptor (D2DR) knockdown mice fail to gain weight or exhibit elevated appetitive motivation in comparison with the wild-type mice within standard environments yet in enriched environments incorporating voluntary exercise facilities, these D2DR knockdown mice expressed markedly lower activity with a rapid increase in obesity compared with the wild-type mice without being receptive of the protective benefit from exercise contingencies [14]. Thus, an underlying mechanism for conceptualizing and treating addictive problems ought to be the reinstatement of a “Dopamine Homeostasis” [15].

It has been found that molecular, cellular and vascular regional brain plasticity [16–18] and neuromorphology [19], involving the medial prefrontal cortex, hippocampus, striatum and amydala, are implicated in both addictive behaviors [20] and the pursuit of physical activity [21]. It has been shown that fitness derived from aerobic exercise at baseline assessments was related selectively to greater thickness in the dorsolateral prefrontal cortex and hippocampus regional volume was associated positively with increased aerobic fitness over time [17]. The notion that sustained physical exercise, possibly rhythmic, may activate opioid systems thereby offering an adjunctive treatment of addictive disorders has been entertained [22, 23]. The integrity of regional brain centers is critical for the expression of exercise interventions: rats with intact medial prefrontal cortical areas showed reduced tendencies to use morphine with accompanying symptomatic (withdrawal) alterations whereas lesioned rats remained unaffected [24, 25]. Certainly, the insertion of exercise intervention for drug abuse patients has produced marked improvements with regard to physical fitness and various aspects pertaining to quality-of-life variables, including daily physical functioning, psychological health and well-being, vitality, social functioning, and general health perceptions as assessed by quantitative measures. Specific physical benefits, indicated by reductions in injuries and muscular pains, decreased weight, and increased vitality with the development of necessary activities of daily living, psychological benefits (i.e. forgetting about everyday problems, improved mood, decreased stress and anxiety), social benefits, and a reduction in craving were estimated through qualitative measures [26]. In the “STimulant Reduction Intervention” program, carried out over nine residential addiction treatment initiatives (USA), a dosed exercise STRIDE intervention increased the mean percentage of abstinence days and levels of abstinence rates among participants [27, 28].

In animal laboratory studies, wheel-running exercise reduced the self-administration of drugs, such as alcohol and nicotine, heroin and cocaine, and 3,4-methylenedioxypyrovalerone (MDPV), in rodents, which in turn were capable of devaluating the ability of the natural reward of exercise to maintain behavior [29–32]. Male rats evidenced a dose-dependent reduction in cocaine-seeking in response to wheel-running [33]; although this effect was evident in female rats also the relationship was not so straightforward. Furthermore, intracellular levels of neurotransmitters are both modulated bi-directionally by drug abuse and addiction [34, 35]. The efficacy of physical exercise under conditions of drug and/or behavioral abuse seems to be connected with the capability of normalizing glutamatergic and dopaminergic signaling events thereby reversing drug-induced changes in chromatin via epigenetic interactions with brain-derived neurotrophic factor (BDNF) in the reward pathway [36]. Exercise alleviates the detrimental effects of negative affective status [37, 38]. Running exercise was found to enhance metabolic rate in rats thereby increasing dopamine availability in the brain with consequential increments to performance [39]. The co-activation dopamine-acetyl choline balance in the context of the nucleus accumbens shell-corticotrophin releasing systems has been shown to affect both reward and affective behavior processes [40]. Morphine exposure during pregnancy increases anxiety-like behavior and increased morphine consumption and drug abuse in the pups [41, 42]. Physical exercise among pregnant rat mothers promoted angiogenesis, neurogenesis, BDNF levels, cognition and reduced anxiety and morphine consumption in the pups [43, 44], as well as in morphine-dependent rats [45]. Exercise schedules during pregnancy for morphine-dependent and non-morphine-dependent rat dams were associated with elevated BDNF concentrations, and increased proliferation and viability of bone marrow stromal cells, vulnerable during addiction [46], in the pups of these dams [42]. Furthermore, voluntary exercise reduced the severity of the anxiogenic-like behaviors, linked to the withdrawal from chronic opiate administration, in both morphine-dependent and morphine-withdrawn rats [47].

The influence of addictive drugs upon the immune system, e.g. reciprocal interaction between the opioid system and the neuroimmune functioning of health systems has been documented [48], incorporating the activation of neuroplastic and neuroinflammatory cascades in the brain [49], implies that potential therapies and interventions, such as physical exercise, that target neuroimmune pathway improvements may be adapted to treat neuropathological and behavioral consequences [50]. Numerous studies have indicated the plethora of health benefits and promotion of effective neuroimmune function resulting from several types of exercise programs over the lifespan of individuals [6, 51–53]. In Wistar rats rendered morphine dependent it was observed that eight weeks of moderate level exercise increased interferon-ɣ and reduced interleukin-17 serum levels [54]. Within the context of morphine withdrawal issues, it was observed that regular swimming exercise (45 min/day, over five days per each week, over the course of 14 or 21 days) reduced the severity of morphine dependence and voluntary morphine consumption with reducing anxiety and depression in morphine-dependent and withdrawn rats [55, 56]. In this regard, it was observed that swimming exercise reduced both conditioned place preference for morphine and behavioral sensitization [57]. Finally, in a rodent model of “drug-craving” it was shown that regular swimming exercise decreased voluntary methamphetamine consumption through the dissipation of anxiety, obsessive-compulsive behaviors, and depression in methamphetamine-withdrawn rats [58].

In conclusion, the present account outlines benefits of physical exercise, independent of type, duration or intensity, pertaining to general health, brain regional, behavioral and somatic integrity and quality-of-life among individuals and laboratory animals stranded in the mire of addictive behaviors, most especially drug abuse. Regular exercise regimes reinstate the modulatory influences of natural rewards through reparation of functional circuits appertaining reward sensitivity, conditioning and cognitive control [59, 60].
